# Supplementary material for: Development of a computer-aided design software for the quantitative evaluation of aesthetic damage
Source: PLoS One. 2019 Dec 18;14(12):e0226322. doi: 10.1371/journal.pone.0226322 (PMC6919621; doi:10.1371/journal.pone.0226322)
Supplement: S1 Code site — 1. (ZIP) [file pone.0226322.s001.zip › [EESC_jr][Projeto]Relatorio_07_21/reports/templates_zip/gentelella-master/vendors/Flot/examples/annotating/index.html]

Flot Examples: Adding Annotations


## Adding Annotations

Flot has support for simple background decorations such as lines and rectangles. They can be useful for marking up certain areas. You can easily add any HTML you need with standard DOM manipulation, e.g. for labels. For drawing custom shapes there is also direct access to the canvas.

Copyright © 2007 - 2014 IOLA and Ole Laursen
